# Tissue Coagulation in Laser Hemorrhoidoplasty – An Experimental Study

**DOI:** 10.1515/med-2020-0027

**Published:** 2020-03-08

**Authors:** Donatas Danys, Julius Pacevicius, Gabija Makunaite, Rolandas Palubeckas, Antanas Mainelis, Narimantas Markevicius, Kestutis Strupas, Tomas Poskus

**Affiliations:** 1 Faculty of Medicine, Vilnius University, Santariskiu 2, LT 08661, Vilnius, Lithuania; 2 Vilnius University Hospital Santaros klinikos, Santariskiu 2, LT-08661, Vilnius, Lithuania; 3 Vilnius City Clinical Hospital, Vilnius, Lithuania; 4 Faculty of Mathematics and Informatics, Vilnius University, Vilnius, Lithuania

**Keywords:** Laser hemorrhoidoplasty, Hemorrhoidectomy, 1470-nm diode laser, Laser coagulation, Perianal tissue, Laser power

## Abstract

**Background:**

Laser hemorrhoidoplasty (LHP) is a new technique for treatment of hemorrhoids. The exact extent of coagulation is not visible at the time of the procedure. There currently is no experimental or clinical data on the subject.

**Objective:**

To evaluate the length of coagulation defect according to power and activation time of 1470nm diode laser on the perianal tissue model.

**Methods:**

Fresh anorectal tissue of twenty-four pigs was used to produce 54 experimental samples. Each sample was randomly assigned to the laser power of 6, 8 and 10 W and 1, 2 or 3-second pulses. The procedure was performed using *Biolitec Ceralas* © diode laser with 1.85 mm optical fiber. The fiber was inserted in a manner, similar to intrahemorrhoidal laser application. Samples were evaluated using low-power and high-power light microscopy by a single pathologist. The length of tissue injury was measured on high-magnification microscopy.

**Results:**

The longest tissue injury (mean 3.93 mm) was caused by the longest laser exposure time (3 sec) with no significant difference between laser power used.

**Conclusions:**

8 W 3-second application of the 1470nm diode laser results in coagulation area approximately 4 mm, and further coagulation should be initiated approximately 5 mm from the first one.

## 1 Introduction

Pain is the most common and severe symptom associated with the surgical treatment of hemorrhoids [1]. It limits the widespread use of excisional hemorrhoidectomy and is the reason for the creation of newer, less invasive treatment methods, such as stapled hemorrhoidopexy and hemorrhoidal artery ligation. However, recurrences after stapled hemorrhoidopexy are significantly more common than after excisional hemorrhoidectomy and long-lasting side-effects, such as tenesmus and fecal incontinence have been reported in a large proportion of patients after the stapled procedure [2]. Hemorrhoidal artery ligation with or without the use of Doppler guidance has been reported to be as effective as a much cheaper rubber band ligation [3]. The search for the optimal treatment of hemorrhoids is ongoing. The success of intravenous laser ablation of large saphenous veins led to experimental and clinical studies of intrahemorrhoidal laser coagulation [4, 5]. Recently introduced technique of laser hemorrhoidoplasty has been successfully tried in patients with all degrees of hemorrhoids. It involves the delivery of laser energy into the interstitial tissue and not into perianal vessels. This leads to retraction of perianal tissues and to the resolution of hemorrhoids with reduced intensity pain of short duration and satisfactory long-term outcomes [6, 7]. We have reported our own experience of laser hemorrhoidoplasty in 229 patients with good short- and long-term outcomes [6]. Similar results were reported by Weyand et al [7]. A randomized controlled trial performed by our group [8] has shown intrahemorrhoidal laser procedure to be more effective than hemorrhoidal artery ligation with a significantly shorter duration of postoperative pain and recovery.

The main peculiarity of laser hemorrhoidoplasty procedure is that the coagulated area is not visible at the time as it is beneath the healthy uninjured mucosal layer. There is no data on the extent of coagulation of perianal tissue with the use of a single pulse of 1470 nm diode laser. It is not known, how many applications must be performed within one hemorrhoid and where the next laser pulse should be applied. The aim of this study was to identify the length of laser coagulation defect after a single laser application based on the duration of application and intensity of laser energy used.

## 2 Materials and methods

The research complied with all relevant national regulations and institutional policies for the care and use of animals.

### 2.1 Study sample

Twenty-four anorectums were excised approximately 5 cm in depth and 2 cm radius around the anus from the freshly slaughtered pigs. Twelve anorectums were taken from male and twelve from female pigs. The experiment was performed within two hours after excision to prevent tissue degradation; all measurements were performed at room temperature, which was 21° C.

Each specimen was assigned a number and divided into two or three parts and mixed up to get 54 samples. Male and female samples were divided equally into each group. Each sample was tagged with the number of the specimen and sex of the pig. For this experiment, 6, 8 and 10 Watt and 1, 2 and 3-second pulses were used. Each sample was randomly assigned to receive the laser power of 6, 8 or 10 W with 1, 2 or 3-second pulse duration. Six specimens were assigned to each laser parameter group, thus 54 experimental interventions were performed.

### 2.2 Experimental intervention

**Figure 1:**
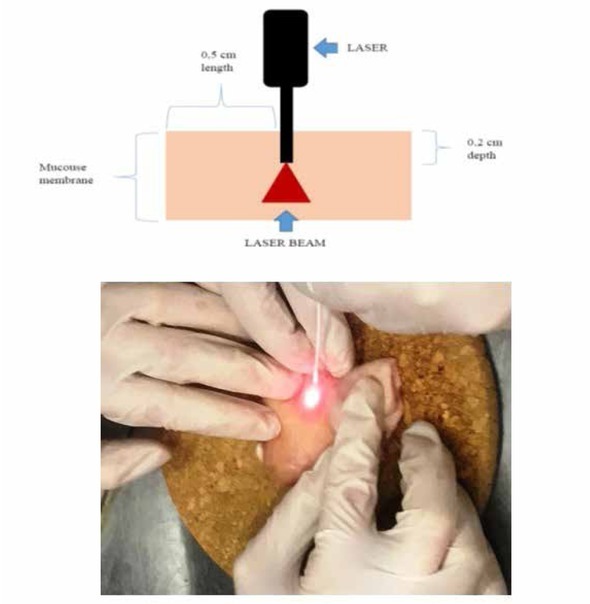
Schematic representation and technique of the experiment

The procedure was performed using “Biolitec“ Ceralas 1470nm diode laser with 6 mm optical fiber. All manipulations were performed by the same team lead by one surgeon (D.D.), experienced in laser hemorrhoidoplasty procedure to avoid variability that may result from different operators. Specimens were affected by different laser parameters. The operator inserted laser fiber perpendicular to the mucosa from outside, approximately 0.5 cm in length and 0.2 cm in depth (Figure 1). The insertion place was marked with a pin for identification during the pathological examination.

Each sample was evaluated after one and ten minutes to identify visual and palpable tissue changes. All changes were recorded.

Specimens were fixed on the plate to prevent shrinkage of tissue before fixing in formalin.

### 2.3 Pathology evaluation

**Figure 2:**
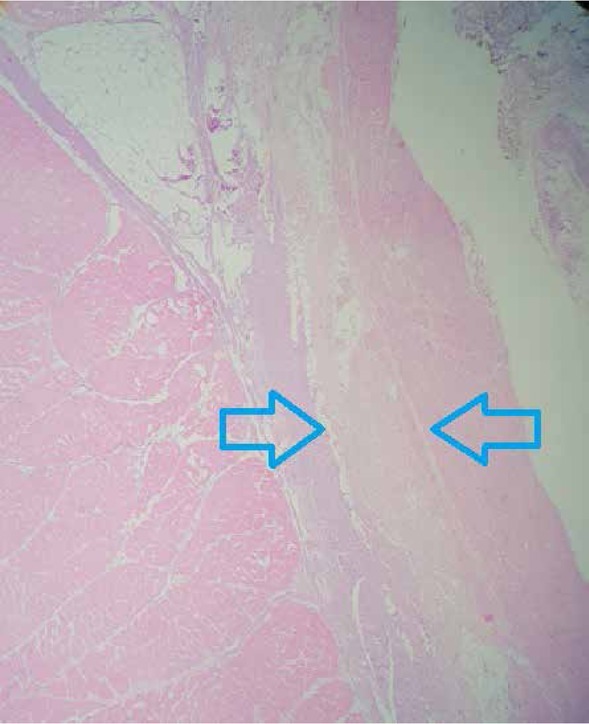
Sections stained with hematoxylin-eosin. Arrows indicate coagulated area

The pathologist was blinded to the parameters of intervention performed on the specimens. Four samples were excised from every specimen- about 2 cm in length, including all layers of the rectal wall for pathological evaluation. Two samples were created by cutting longitudinally to the direction of the laser insertion site; sections were performed next to each other. Two other samples were created by cutting vertically to the direction of laser insertion place, about 0.5- 1 cm away from the terminal site of laser coagulation. The area of the evaluation was 4 cm in longitudinal sections and 2 cm in vertical sections. All sections were prepared in the tissue processor, paraffin blocks of tissues were created. Each block was cut into sections of 3 μm in thickness and conventional hematoxylin-eosin staining was performed. Sections were evaluated under low- and high-power light microscopy by the same pathologist. Sections were observed at different magnifications (40× and 100×) for the measurement of the length of tissue injuries (Figure 2). The extension of tissue injuries was measured if the tissue was damaged through in one of four histopathological sections. All measurements were made using a ruler of a microscope.

All data were collected for evaluation. Statistical analysis was performed with *SAS On Demand for academics*, using multiway ANOVA, two-way ANOVA and Chi-square tests.

## 3 Results

The results of the extent of soft tissue coagulation injury in the specimens are presented in table 1.

**Table 1:** Data of tissue damage, palpable and visual changes in different groups.

Laser power (Watts)	Exposure time (Sec)	Average of tissue damage (mm) +/- standart deviation (mm)		Medians of tissue damage (mm)	Palpable changes	Visible changes
6	1	1,62 +/- 1,99		0,85	0	0
6	2	1,78 +/- 1,06		2,0	0	0
6	3	2,78 +/- 1,97		2,95	0	0
8	1	0,63 +/- 0,99		0,15	0	0
8	2	2,67 +/- 1,88		2,7	0	0
8	3	3,15 +/- 3,18		2,75	1	0
10	1	1,12 +/- 2,5		0	0	0
10 10	2 3	1,72 +/- 1,64 5,85 +/- 3,87		1,55 5,6	1 1	0 0

Soft tissue damage mostly depends on laser exposure time (p= 0.0027), with no significant difference between laser power used (p= 0.5086). Extended laser exposure time is a cause of longer tissue damage. In the 1-second group, tissue damage on average reaches 1.12 mm, in 2 seconds group – 2.06 mm and in 3 seconds group – 3.93 mm.

The depth of tissue damage within various laser power groups (6W, 8W, and 10 W) varies on average from 2.06 mm to 2.89 mm, with no significant difference between groups (p= 0.5086).

The difference in length of tissue damage between different power intensity groups was not statistically significant (p>0.05).

No changes were palpable within 1 minute after laser application in all groups. Palpable changes10 minutes after laser application were related to the power of laser exposure: 6W – 0%, 8W – 33.3%, 10W – 66.7% (p= <0.0001). Longer laser exposure time caused higher rate of palpable tissue changes after 10 minutes: 1 sec – 11.1%, 2 sec – 38.9%, 3 sec – 50,0% (p= 0.039).

No visible changes were noticed during the visual evaluation of samples after 1 and 10 minutes.

## 4 Discussion

**Figure 3:**
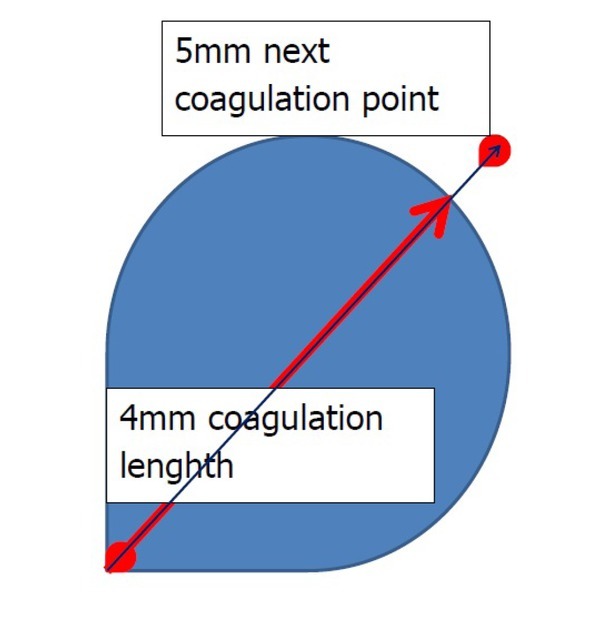
Schematic recommendation for coagulation during LHP

In this study, we found that the 4 mm area of the anorectal submucosa is coagulated after the 3-second application of the laser. This is very important clinically – additional coagulation within the same area would only produce further coagulation and necrosis, thus the coagulation should start in an area at least 5 mm from the initial spot (Figure 3). We also found that the length of coagulation depends significantly more on time of application than on the power of application. Palpable changes occur only after 10 minutes after coagulation and they are felt more frequently with increasing power of coagulation and less with increasing time of laser application.

The main strength of our study is the controlled nature of the experiment. External variables were controlled: groups of animals, used in this research, are homogeneous (by sex, age and weight), experimental conditions, air temperature, time to prepare and data collecting time were the same. A single surgeon performed all procedures to avoid variation as well, single pathologist performed the pathological study of the specimens. Moreover, the porcine tissue model has been chosen, as the anatomy and physiology of the digestive system of the pig, especially of the large intestine, are similar to human [9]. The pathologist was blinded to the settings of the intervention performed on the specimens. This experimental research design is easily reproducible. However, there are some weaknesses in our research. Live and vascularized tissue may produce slightly different areas of coagulation necrosis due to the cooling effect of circulating blood, however, our study clearly identifies the minimal area, in which additional coagulation would be hazardous.

The first experimental model of diode laser application in monkeys was performed by Plapler [10]. However, the aim of his study was to evaluate the effectiveness of diode laser in the treatment of hemorrhoids. The laser power used was 1-2 W and the exposure time was 1 s. Their study showed that diode laser energy delivered into the hemorrhoids led to their complete resolution. But the correlation between soft tissue injury and the exposure time and laser power was not investigated [4]. The first human study of intrahemorrhoidal laser was performed with 15 patients. Laser power, used in this study, was 5 W. Major complication was burn lesions and it was noticed that some adjustments must be made to prevent it. Intrahemorrhoidal laser effects on perianal tissue, which depend on laser power and exposure time, have not been studied until now. In addition, even though humans and pigs share very similar anatomy and tissue structure, we couldn‘t find any reliable verification, that laser would cause the same reactions on humans. Subjective factors like pain, discomfort, and soreness also play an important role in clinical practice and couldn’t be investigated in this experimental stage.

This study clearly identifies the length of coagulation after a single application in commonly used laser power and duration settings. In this area, additional laser application is hazardous and will only result in tissue carbonization and burn injury.

## 5 Conclusion

8 W 3 second application of the 1470nm diode laser results in a coagulation area approximately 4 mm and further coagulation should be initiated approximately 5 mm from the first one. Palpable changes of perianal tissues were detected 10 minutes after laser exposure time and no visible changes could be seen in the specimens.
